# Kinetic Model for Describing the Spring Effect and Incomplete Dissolution in Low-Solubility Drug Formulations Based on the Quality-by-Design (QbD) Approach

**DOI:** 10.3390/pharmaceutics18101334

**Published:** 2026-10-21

**Authors:** László István Orosz, István Fábián, József Kalmár

**Affiliations:** 1 HUN-REN-DE Mechanisms of Complex Homogeneous and Heterogeneous Chemical Reactions Research Group, Department of Inorganic and Analytical Chemistry, University of Debrecen, Egyetem Tér 1, H-4032 Debrecen, Hungary; 2 Doctoral School of Chemistry, University of Debrecen, Egyetem Tér 1, H-4032 Debrecen, Hungary

**Keywords:** drug release, amorphous solid dispersion, spring effect, kinetic modelling

## Abstract

**Background/Objectives:** Enhancing the bioavailability of active pharmaceutical ingredients (APIs) with low solubility –classified under the Biopharmaceutics Classification System (BCS) as class II and class IV –is a major challenge in pharmaceuticals development. Stabilizing APIs in amorphous form significantly increases their apparent solubility but results in complex dissolution profiles characterized by the “spring effect,” a temporal high concentration peak during dissolution. These dissolution profiles are very challenging to be quantitatively evaluated due to the lack of appropriate mathematical models compatible with a Quality-by-Design (QbD) approach. **Methods:** A kinetic model was developed by considering the mechanistic aspects of the process. The model postulates three stages of the dissolution: hydration and dissolution of the amorphous API within the carrier, diffusion of the dissolved API into the medium, and equilibrium-driven interactions between the dissolved API and its thermodynamically stable crystalline form. **Results:** This model provides insights into release acceleration, slow final dissolution, and the spring effect, and elucidates scenarios where complete dissolution cannot be achieved due to the absence of sink conditions. While microscopic molecular phenomena –such as liquid–liquid phase separation (LLPS) and polymeric crystallization inhibition –are extensively documented in the academic literature, their individual isolated parameters are inherently unobservable under routine pharmaceutical industrial and GMP testing conditions. To bridge this gap, the developed model establishes a robust, apparent kinetic framework that fits macroscopically observable dissolution profiles. **Conclusions:** This method provides a robust framework for interpreting drug release profiles and optimizing formulation development using a QbD approach, specifically supporting regulatory requirements and batch-to-batch consistency evaluations across similar polymer-matrix systems. The model enables the determination of kinetic parameters such as *t*_max_ and *c*_max_, and explains their dependence on the actual pharmaceutical formulation.

## 1. Introduction

Enhancing the bioavailability of poorly water-soluble active pharmaceutical ingredients (APIs)—classified under the Biopharmaceutics Classification System (BCS) as class II (high permeability, low solubility) and class IV (low permeability, low solubility)—is a major challenge in modern pharmaceutics, because their limited solubility and/or permeability complicate oral delivery and reduce therapeutic efficacy [1,2,3]. To address these limitations, various formulation strategies have been employed, including the use of thermodynamically non-stable amorphous forms such as amorphous solid dispersions (ASDs), or administering APIs in high concentrations in liquid dispersions. The stabilization of these metastable systems remains a key formulation challenge [4,5,6,7]. Among emerging experimental technologies, aerogels have gained increasing attention as solubility-enhancing excipients [8,9,10,11]. Due to their high porosity and surface area, aerogels can incorporate APIs in amorphous form, resulting in significantly enhanced dissolution profiles and bioavailability [8,9,10].

The use of amorphous formulations results in unique drug release kinetics. When APIs are present in amorphous form, they tend to dissolve rapidly, often reaching a supersaturated state. This phenomenon, frequently referred to as the “spring effect,” is characterized by an initial sharp increase in drug concentration followed by crystallization (precipitation) depending on the system’s thermodynamic and kinetic characteristics [12,13,14,15,16]. In many cases, especially at higher dose strengths, this also results in incomplete dissolution—commonly observed as “less than 100% dissolution”—either due to precipitation or non-sink conditions [5,13,17,18].

To mechanistically describe complex dissolution behaviors—such as the spring effect, early acceleration, and incomplete dissolution—a multi-stage kinetic model was developed. This model divides the process into sequential stages: hydration and release of the amorphous API from the carrier, diffusion into the medium, and equilibrium-driven transformation into the stable crystalline form [19,20,21,22]. Crucially, it also accounts for cases where full dissolution is not achieved due to crystallization or saturation constraints [5,13,22,23].

Empirical multi-stage kinetic approaches have been explored in previous studies linking supersaturation and crystallization behavior, as well as describing crystallization kinetics following the dissolution of the amorphous drug [5,12,13,14,22,23].

While these previous models have described supersaturation and recrystallization phenomena, the present model advances these efforts by quantitatively describing all key dissolution steps—including hydration, diffusion, and crystallization—into a unified mathematical structure [19,22,24,25]. This allows for the simultaneous estimation of dissolution kinetic parameters such as *t*_max_ and *c*_max_, and their direct correlation with formulation variables. This has not been comprehensively integrated into a unified framework before our work [19,24,25]. Such a kinetic model supports the Quality-by-Design (QbD) approach, enabling the rational optimization of drug release [26,27,28].

It is well-established that the dissolution of ASDs and the subsequent “spring effect” involve intricate, sub-micron molecular mechanisms. When the drug concentration exceeds its amorphous solubility, liquid–liquid phase separation (LLPS) frequently occurs, generating drug-rich nano-droplets or colloidal species. Furthermore, the subsequent precipitation or crystallization from these supersaturated states is governed by a multi-step interplay of nucleation, crystal growth, and polymer-mediated crystallization inhibition. Polymers typically prolong supersaturation (the “parachute effect”) by altering solution viscosity, reducing molecular mobility, or interacting directly with drug surfaces to impede crystal propagation.

However, from a practical regulatory and Quality by Design (QbD) perspective within industrial Good Manufacturing Practice (GMP) settings, isolating these highly interdependent microscopic variables during standard dissolution testing is neither technically feasible nor practical. Routine quality control relies on macroscopic, apparent concentration–time profiles. Therefore, rather than attempting to resolve hardly observable molecular descriptors, the objective of this work is to present a unified, lumped mathematical model. This approach successfully segregates drug-formulation-specific behavior and provides rate constants capable of quantitatively supporting biowaiver justifications and validating the robustness of similar pharmaceutical formulations.

## 2. Material and Methods

### Definition of the Physical and Chemical Background of Modeling

The dissolution of a solid pharmaceutical dosage form can be described by a series of consecutive, simplified physical processes [19,20,21,22]. It is useful to examine the process under pharmaceutical GMP (Good Manufacturing Practice) conditions, where only a very limited number of physical quantities is observed with precision, and the amount of data generated during such dissolution testing is often limited [26,27,28]. Therefore, it is practical to conceptualize the dissolution process in its simplest form, which necessitates the design of a simplified kinetic model [19,24,25]. The GMP dissolution process can be most comprehensively monitored using a Pharmacopoeia UV Online Dissolution Testing Apparatus [18,29].

**Figure 1:**
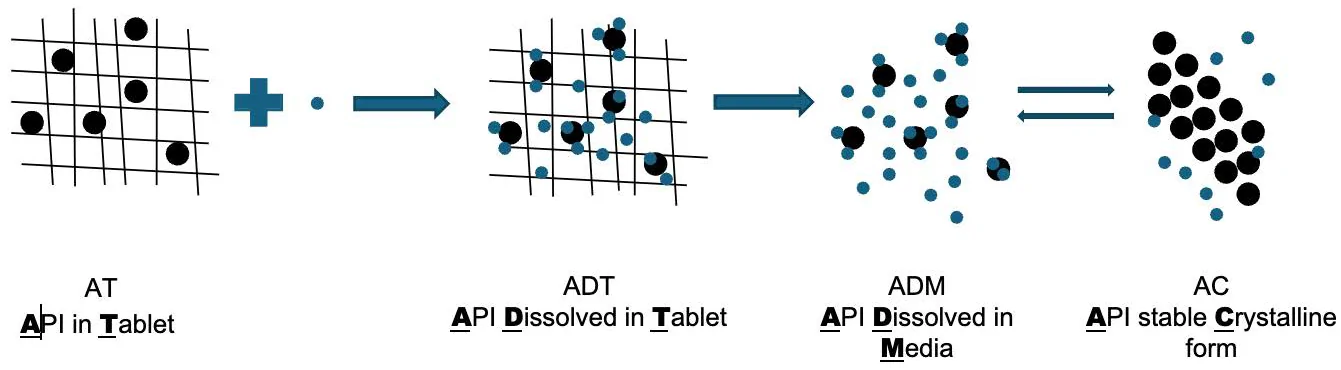
Conceptual scheme of the model describing the dissolution process of the API. The model distinguishes four states: solid API in the pharmaceutical formulation (AT), dissolved API within the matrix (ADT), dissolved API in the medium (ADM), and stable crystalline API precipitated from solution (AC). Solid arrows denote unidirectional mass transfer processes, while the reversible arrow indicates a heterogeneous equilibrium. The schematic provides the structural basis for the system of differential equations introduced in Section 3.

Our approach focuses on the following aspects. The dissolution process starts with the hydration of the active pharmaceutical ingredient (API) in the solid dosage form, transitioning it into a state similar to its dissolved form (Figure 1). In this hydrated state, the API remains within the wetted matrix [4,5,6,7]. The specific physical form of the API in the original formulation prior to hydration—whether crystalline in a specific polymorph form or amorphous—is taken into account in the model [4,5,23,30].

Once the hydrated API is present in the system, a second process begins, during which the hydrated API molecules transition (diffuse) into the dissolution medium [19,20,21]. This process is monitored using UV–VIS spectrophotometry or chromatographic methods [18,29]. If the solubility of the API is lower in the dissolution medium than the maximum concentration of the available API, crystallization begins to take the thermodynamically stable form [5,13,17,22,23]. This phenomenon is an equilibrium process, where the thermodynamically stable crystalline form precipitates from the solution and forms a suspension in heterogeneous equilibrium with the saturated solution [5,13,17]. Based on the relative rates of these processes, the concentration of the dissolved API in the medium can show distinct and unique temporal profiles, as discussed in the next sections of this paper [8,13,14,15,16,22].

Crucially, while the majority of modern clinical formulations utilize polymer-based molecular dispersions where the dissolved carriers exert a crystallization-inhibiting “parachute effect”, our modeling framework intentionally avoids the explicit inclusion of time-dependent polymer dissolution curves or concentration variables. To address this, the present model segregates the “parachute effect” by treating each specific formulation as an independent mathematical entity characterized by its own distinct set of apparent kinetic constants. Consequently, any variation in the formulation properties, composition, or drug-to-polymer ratio is directly reflected in the fitted macro-constants rather than requiring explicit multi-component tracking. This operational simplification is beneficial for practical regulatory utility and batch comparability, providing a robust mathematical tool for regulatory bioequivalence (biowaiver) assessments without the risk of numerical over-parameterization.

## 3. Mathematical Description of the Model

The mathematical description of the multi-step drug release mechanism depicted in Figure 1 is as follows.

(1) $$\mathrm{AT}\overset{k_{1}}{\to }\mathrm{ADT}\overset{k_{2}}{\to }\mathrm{ADM}\overset{k_{3}}{\underset{k_{4}}{⇌}}\mathrm{AC}$$

(2) d[AT]/dt = −*k*_1_ × [AT]

(3) d[ADT]/dt = *k*_1_ × [AT] − *k*_2_ × [ADT]

(4) d[ADM]/dt = *k*_2_ × [ADT] − *k*_3_ × [ADM] + *k*_4_ × [AC]

(5) d[AC]/dt = − *k*_4_ × [AC] + *k*_3_ × [ADM]

The change in the concentration of the hydrated API in the matrix (ADT) is governed by the interplay of two processes: its formation via the hydration of the dry API (AT), and its release into the dissolution medium.

Equation (4) describes the change in the concentration of the dissolved API in the dissolution medium (ADM). This form represents the bioavailable fraction of the API. Its concentration increases due to release from the hydrated API from the matrix phase (ADT), and decreases via crystallization into the thermodynamically stable form (AC), when the ADM concentration exceeds the solubility limit.

Equation (5) characterizes the rate at which the equilibrium is reached between the dissolved API (ADM) and its precipitated crystalline form (AC), as discussed in Section 4.1 and Section 4.2.

## 4. Solution of the Differential Equation System

**Figure 2:**
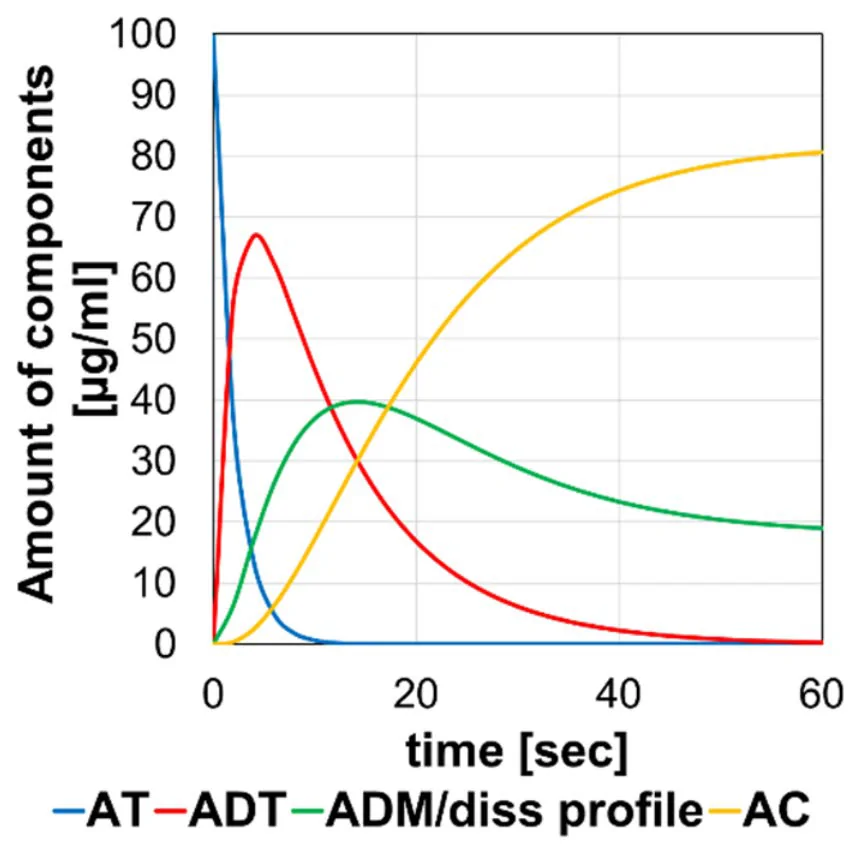
Concentration of the different forms of the API vs. time calculated using Equation (9). The dissolution profile is equivalent to the temporal concentration of ADM. Parameters: AT (*t* = 0) = 100 µg/mL, ADT (*t* = 0) = ADM (*t* = 0) = AC (*t* = 0) = 0 µg/mL, *k*_1_ = 0.5 s^–1^, *k*_2_ = 0.1 s^–1^, *k*_3_ = 0.09 s^–1^, *k*_4_ = 0.02 s^–1^, *S* = 18 µg/mL (solubility).

The system of four linear ordinary differential equations (ODEs) describing the dissolution process can be efficiently solved using matrix exponential methods [19,24,25]. By expressing the system in matrix form, the solution is obtained as the product of the matrix exponential (Equation (7)) and the initial condition vector (Equation (8)), providing a direct and computationally tractable approach [19,24]. The temporal evolution of the species concentrations is illustrated in Figure 2, based on the analytical solution defined by the matrix exponential (Equation (9)) in case *k*_2_ > *k*_3_ (*vide infra*) [19,20,22].

This characteristic yields a temporarily supersaturated API solution. In other words; if the crystallization (precipitation) process is slower than the release of the API from the matrix, the dissolved API concentration (ADM) shows a clearly detectable maximum as a function of time [12,13,14,15,16,22].

(6) $$\frac{d\underline{C}}{\mathrm{dt}}=\underline{\underline{k}}\underline{C}$$

(7) $$\underline{C_{0}}=\left[\begin{array}{c} {\mathrm{AT}}_{0}\\ {\mathrm{ADT}}_{0}\\ {\mathrm{ADM}}_{0}\\ {\mathrm{AC}}_{0} \end{array}\right];\;\underline{\underline{k}}=\left[\begin{array}{cccc} {-k}_{1} & 0 & 0 & 0\\ k_{1} & {-k}_{2} & 0 & 0\\ 0 & k_{2} & -k_{3} & k_{4}\\ 0 & 0 & k_{3} & {-k}_{4} \end{array}\right];\;\underline{C_{t}}=\left[\begin{array}{c} {\mathrm{AT}}_{t}\\ {\mathrm{ADT}}_{t}\\ {\mathrm{ADM}}_{t}\\ {\mathrm{AC}}_{t} \end{array}\right]$$

(8) $$\underline{C_{t}}=\mathrm{expm}\left(\underline{\underline{k}}t\right)\times \underline{C_{0}}$$

(9) $$\mathrm{expm}\left(\underline{\underline{M}}\right)=\sum_{n=0}^{\infty }\frac{1}{n!}{\underline{\underline{M}}}^{n}$$

### 4.1. Model Scope and Validity Range

The proposed kinetic model describes the transient dissolution–transformation process of the API from the intact pharmaceutical formulation state up to the establishment of heterogeneous solubility equilibrium.

Importantly, the model is formulated to describe the dynamic approach to reach the solubility equilibrium rather than the mechanistic details of the equilibrium state itself. Similar approaches have been described in the literature [13,14,21,22]. Once thermodynamic equilibrium between the dissolved and crystalline phases is reached, the net mass flux between ADM and AC becomes zero. At this stage, the system is governed by the thermodynamic constraints [5,13,17,23].

The present formulation provides a compact representation of the experimentally observable concentration profiles within this transient regime [19,24,25].

To clarify the mechanistic limits of this framework, we explicitly acknowledge that the dissolution and subsequent de-supersaturation of ASDs involve highly complex, sub-micron phenomena. In the early stages of the “spring effect”, when the local drug concentration exceeds its amorphous solubility, liquid–liquid phase separation can occur, leading to the formation of drug-rich colloidal nano-droplets or phase-separated species. Furthermore, subsequent crystallization is inherently a multi-step process comprising nucleation, crystal growth, and polymer-mediated inhibition governed by viscosity and molecular mobility.

However, from a Quality by Design (QbD) and routine industrial quality control (QC) perspective, these individual microscopic steps are fundamentally unobservable and technically non-separable under standard pharmacopoeia testing configurations. Therefore, the proposed model does not resolve individual thermodynamic parameters or independent multi-step crystallization pathways. Instead, the crystallization rate constant (*k*_3_) is defined as an apparent, lumped global kinetic parameter that quantifies the combined, cooperative macroscopic outcome of these concurrent processes.

Similarly, the quantified dissolved pool (*ADM*) represents the apparent dissolved concentration, encompassing both truly molecularly dissolved API and any sub-micron colloidal or phase-separated drug species capable of passing through standard analytical filtration boundaries [30,31]. Because non-sink or near-non-sink supersaturation studies are heavily interdependent with experimental hydrodynamics, these fitted kinetic constants should be interpreted as apparent, system-dependent parameters rather than intrinsic formulation invariants. They reflect the combined performance of the matrix under the specified dissolution methodology, medium volume, agitation speed, and sampling procedures. While this operational abstraction limits the model’s capacity to predict molecular transitions, it ensures highly accurate, numerically stable replication of macroscopic dissolution profiles and provides a robust, standardized mathematical engine to support matrix comparison and biowaiver criteria in regulatory submissions.

The distinction between transient kinetic modeling and equilibrium thermodynamic description is intentional and ensures numerical robustness within the experimentally accessible time domain [19,20,24].

Within the investigated experimental conditions, the model reproduces the observed concentration–time profiles with high accuracy until equilibrium is reached, as demonstrated later in Section 7.

### 4.2. Relationship Between Parameters and Physical Processes

The rate constant *k*_1_, is associated with the hydration and the water uptake of the pharmaceutical product. This relationship can be approximated as a simplified process: once the product absorbs water in a small volume, the amorphous API rapidly transitions into its hydrated (dissolved) form within the matrix [4,5,6,7].

The *k*_2_ rate constant is associated with mass transfer processes, during which the hydrated form of the API transitions (diffuses) from the wet product matrix into the dissolution medium. Based on current observations and considerations, *k*_1_ >> *k*_2_ [19,20,22].

The *k*_3_ and *k*_4_ rate constants are related to the precipitation and dissolution of the thermodynamically stable dissolved and crystalline forms of the API, respectively, thus convey information on the rate at which the solubility equilibrium is reached [5,13,17,22,23]. In our framework, we purposely define *k*_3_ as an apparent, lumped global kinetic constant, which represents the cooperative, macroscopic velocity of the entire de-supersaturation cascade under the specific experimental setup.

Consequently, the fitted parameters (*k*_1_ to *k*_4_) must be interpreted as apparent, system-dependent parameters rather than intrinsic, thermodynamic formulation invariants.

The most interesting case is when *k*_2_ > *k*_3_, which condition yields a maximum in the [ADM] vs. time curve that exceeds the solubility of the crystalline API (*S*) [12,13,14,15,16].

However, when equilibrium is reached, the equilibrium concentration of ADM is defined by the solubility limit (*S*) (Equation (10)) and not by the rate constants *k*_3_ and *k*_4_ [5,13,17].

(10) [ADM]_∞_ = *S*

The total amount of API present in the system is conserved, and the final crystalline content [AC]_∞_ can be expressed as:

(11) [AC]_∞_ = [AT]_0_ + [ADT]_0_ + [ADM]_0_ + [AC]_0_ − *S*

This mathematical formulation guarantees that the system approaches the solubility limit *S*, thereby ensuring thermodynamic consistency at the macroscopic level [19,20,24]. The parameter *S* does not appear explicitly in the differential equations, because the system approaches equilibrium asymptotically through the rate constants *k*_3_ and *k*_4_. Once thermodynamic equilibrium between the dissolved and crystalline phases is reached, the net mass flux becomes zero.

It is not necessary for the model to specify concrete pH values for the dissolution medium, but it is very important that constant pH is maintained during the dissolution test, i.e., maintaining the experimental conditions during a specific run. The model is sensitive to the pH change and detects this if the multiple dissolution tests to be compared are run at different pH conditions, because the rate constants are different at different pH values.

#### Relationship Between Initial Conditions and Physical Processes

During the development of a pharmaceutical dosage form, the particle size distribution of the active pharmaceutical ingredient (API) and the variability of crystal forms are typically maintained within a very narrow range [1,3,28]. If multiple polymorphic forms are present in the product, they often behave differently in terms of the dissolution rate [4,5,23,30]. The described model is designed to represent the behavior of a final pharmaceutical product containing an API in a narrow particle size distribution, either in a homogeneous crystalline (specific polymorph) form or amorphous state [4,5,6,7]. Naturally, the amount of API in the product has a significant effect on the dissolution profile [5,12,13,17].

At the same time, estimating the rates of the processes suggests that the model is capable of predicting the behavior of more complex pharmaceutical formulations [19,20,22]. It is a potential risk that the amorphous form may transform into a thermodynamically stable crystalline form during storage [6,7,23,30]. In this scenario, the model can simulate the phenomenon where part of the API is in the amorphous form and another part is in the thermodynamically stable crystalline form.

To describe this situation in a regulatory-consistent manner, the concept of label claim is introduced. While strength refers to the actual amount of API present in the dosage unit (mg/tablet), the label claim denotes the declared API content printed on the product label, which serves as the reference value for dissolution normalization and biowaiver evaluation. In systems where both amorphous (AT) and crystalline (AC) API fractions are initially present, the declared API amount corresponds to the sum of these solid-state contributions, expressed as: [Label Claim] = [AT]_0_ + [AC]_0_. This phenomenon significantly impacts the dissolution curves, because the presence of the slowly and poorly soluble crystalline form significantly changes the dissolution profile [5,13,17,23].

### 4.3. Impact of Dose Strength and Label Claim on Dissolution Kinetics

**Figure 3:**
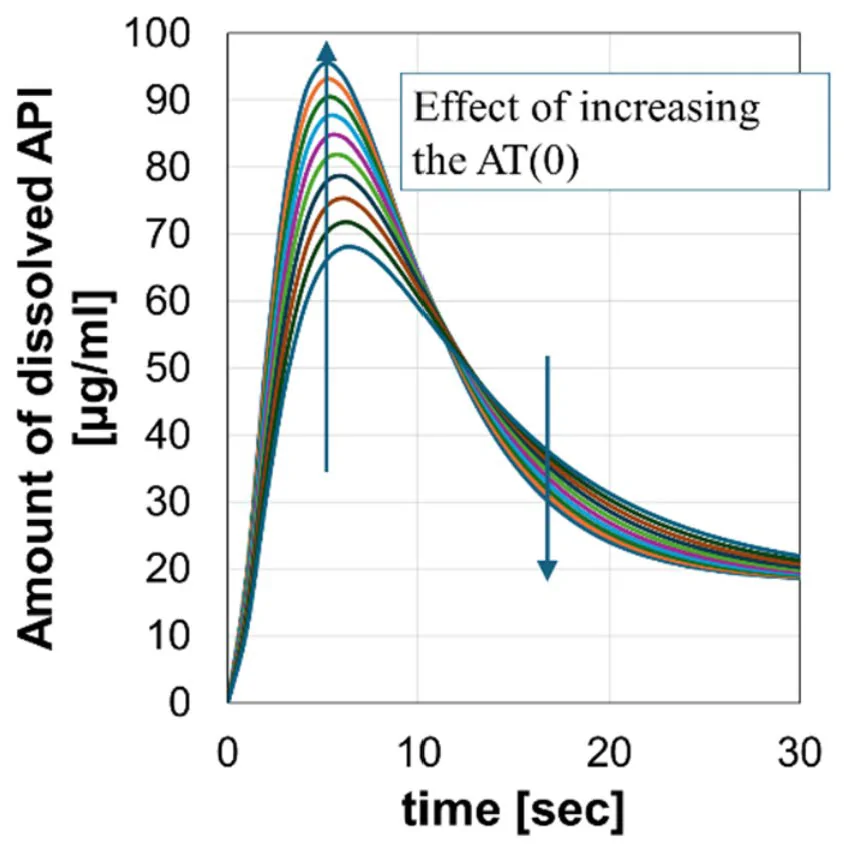
The change in the dissolution profiles with changing pharmaceutical dose strength. Parameters: AT (*t* = 0) = 100–200 µg/mL, ADT (*t* = 0) = ADM (*t* = 0) = AC (*t* = 0) = 0 µg/mL, k_1_ = 0.5 s^–1^, k_2_ = 0.1 s^–1^, k_3_ = 0.09 s^–1^, k_4_ = 0.02 s^–1^, S = 18 µg/mL (solubility).

It is challenging to compare different pharmaceutical products based solely on parameters such as *t*_max_ (time to reach maximum concentration), *c*_max_ (maximum concentration), and equilibrium API solubility (*S*). For example, if a product with a higher API content is used, it may result in higher *c*_max_, as well as shifted *t*_max_. In the case of higher API content, the rate of crystallization and precipitation of the released drug may also be faster, as seen in Figure 3. This distortion can make it challenging to accurately evaluate and compare products that have different initial active ingredient contents, even if other conditions are kept constant.

To resolve this ambiguity and align the model with regulatory bioequivalence and Quality by Design (QbD) requirements, a strict distinction must be made between the analytical *assay* (the total chemical amount of API per dosage unit) and the *label claim* (the declared, standardized nominal dose printed on the product label). Any absolute excess in total drug mass is captured by standard assay testing. However, from a regulatory biowaiver and quality control perspective, dissolution profiles must be normalized against the label claim to assess similarity (e.g., using fit factors or kinetic modeling).

Introducing the label claim as a fixed reference value in our model serves a dual purpose. First, it eliminates dose-dependent scaling artifacts, allowing the direct comparison of the apparent kinetic constants (*k*_1_ to *k*_4_) across different product strengths. Second, it provides a mathematically rigorous tool to simulate and quantify solid-state aging or manufacturing non-uniformity—specifically, when a portion of the API inadvertently transitions from the amorphous form into the thermodynamically stable crystalline form prior to dissolution. In such a scenario, while the total API content (the *assay*) remains 100% compliant, the fraction of the API that is amorphous and capable of driving the “spring effect” is significantly reduced. By fixing the total API (crystalline + amorphous) content according to the label claim, the model can separate the kinetic contribution of the remaining amorphous fraction from the crystalline solubility. This capability provides a distinct advantage over model-independent approaches, transforming the model into a predictive regulatory tool for batch-to-batch consistency and stability evaluations.

As a result, it may not be appropriate to rely solely on model-independent procedures for evaluating products with a spring effect. Alternative approaches that take into account the specific characteristics of such products may be necessary. For example, conducting controlled experiments with varying active ingredient content and carefully analyzing the resulting data may provide more meaningful insights. Additionally, it may be beneficial to consider other factors, such as the mechanism of action of the active ingredient and the intended use of the product, when evaluating and comparing products with a spring effect.

### 4.4. Effect of Solid-State Recrystallization

ASD products are prone to recrystallization of the amorphous API into its thermodynamically stable crystalline form, often triggered by environmental factors such as exposure to moisture [15,27,32]. This transformation can significantly reduce bioavailability, because the solubility of the crystalline form is low [33].

**Figure 4:**
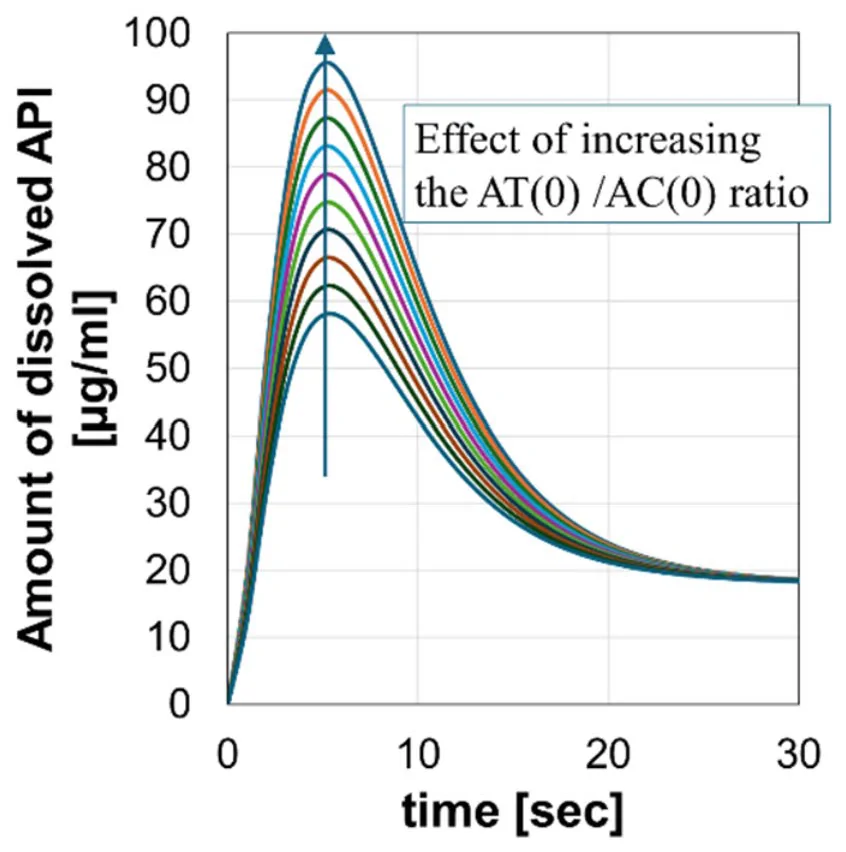
Stability and recrystallization study; effect of increasing the AT(0)/AC(0) ratio; AT (*t* = 0) + AC (*t* = 0) = 200 µg/mL, AT (*t* = 0) = 110–200 µg/mL, AC (*t* = 0) = 90–0 µg/mL, ADT (*t* = 0) = ADM (*t* = 0) = 0, k_1_ = 0.5 s^–1^, k_2_ = 0.1 s^–1^, k_3_ = 0.09 s^–1^, k_4_ = 0.02 s^–1^, S = 18 µg/mL (solubility).

The kinetic model presented here allows the simulation of such recrystallization effects by adjusting the initial ratio of amorphous ([AT]_0_) and crystalline ([AC]_0_) API. Figure 4 illustrates how changing [AT]_0_/[AC]_0_ ratio within the same dose strength alters the dissolution profile. The observed trend is primarily driven by the reduced amount of amorphous API available for rapid hydration and release.

Importantly, the presence of crystalline API at *t* = 0 does not directly slow down the initial hydration step, but it reduces the proportion of API that can undergo fast dissolution. As a result, the initial rate of concentration increase (spring effect) diminishes, and the system approaches the solubility limit more gradually (Figure 4).

## 5. Sensitivity of the Model Regarding Kinetic Parameters

### 5.1. Effect of Values of k_1_ and k_2_

The effects of *k*_1_ and *k*_2_ on the dissolution of an active ingredient are similar. As *k*_1_ and *k*_2_ increase, the solubilization and release of the active ingredient is faster, which in turn leads to a reduction in *t*_max_ and an increase in the maximum concentration (*c*_max_). The dissolution rate increases as the concentration of the API increases, resulting in a sharp increase in the dissolution rate at higher concentrations [34].

Regardless of the specific values of *k*_1_, *k*_2_, *k*_3_, *k*_4_, the dissolution rate increases in the initial part of the drug release process. Therefore, it is unnecessary to simplify this behavior by introducing a lag time (*t*_lag_). This initial acceleration can be beneficial for optimal bioavailability [6,16].

The specific values of *k*_1_, *k*_2_, *k*_3_, *k*_4_ vary depending on the specific API and the applied formulation. Additionally, other factors, such as pH and temperature, can also influence solubility and dissolution. However, when studying a series of strongly related pharmaceutical formulations, these effects can be treated as incorporated into the *k* values.

For extended-release behavior, if the analytical system can detect low concentrations, the acceleration phase can be modeled and parameterized. The initial acceleration phase in the dissolution curve is particularly important when the API is rapidly absorbed biologically, because in such cases, absorption occurs at the beginning of the release process, significantly impacting the blood plasma concentration profile [35].

### 5.2. Effect of Values of k_3_ and k_4_

In the model describing crystallization and solubility, the rate constants *k*_3_ and *k*_4_ are linked through the reversible transformation of the dissolved API (ADM) into its crystalline form (AC). We propose that as the concentration of the dissolved API increases, the rate of crystallization also increases, leading to faster formation of the solid phase. Until reaching the solution equilibrium, both dissolution and crystallization processes accelerate when more of the dissolved and the crystalline API is present, although the exact mechanism is not resolved by the current model.

When the rate constant *k*_3_ is higher than *k*_2_, the dissolution profile ceases to display an easily detectable maximum, which also makes it hard to decide whether the spring effect is operational or not.

## 6. Modeling the Dissolution Profiles of Felodipine and Indomethacin Across Diverse Formulations

To evaluate the versatility and robustness of the proposed multi-stage kinetic framework, published experimental dissolution data reported by Alonzo et al. were fitted using Equations (6)–(9) [5]. The dataset encompasses two model poorly water-soluble APIs indomethacin and felodipine tested under widely varying formulation parameters, including the presence of pre-dissolved crystallization-inhibiting polymers (polyvinylpyrrolidone, PVP; hydroxypropyl methylcellulose, HPMC), different temperatures (25 °C and 37 °C), and a wide variation of initial API loadings (100–5500 μg/mL).

The model was fitted to selected dissolution profiles covering distinct supersaturation and precipitation behaviors. The purpose was to test whether the model reliably describes different kinetic regimes and yields physically meaningful parameters, rather than simply fitting every curve empirically. Unlike empirical models, the fitted rate constants (*k*_1_–*k*_4_) provide mechanistic information on hydration, drug diffusion, and precipitation covering a broad range of conditions to benchmark the model.

**Figure 5:**
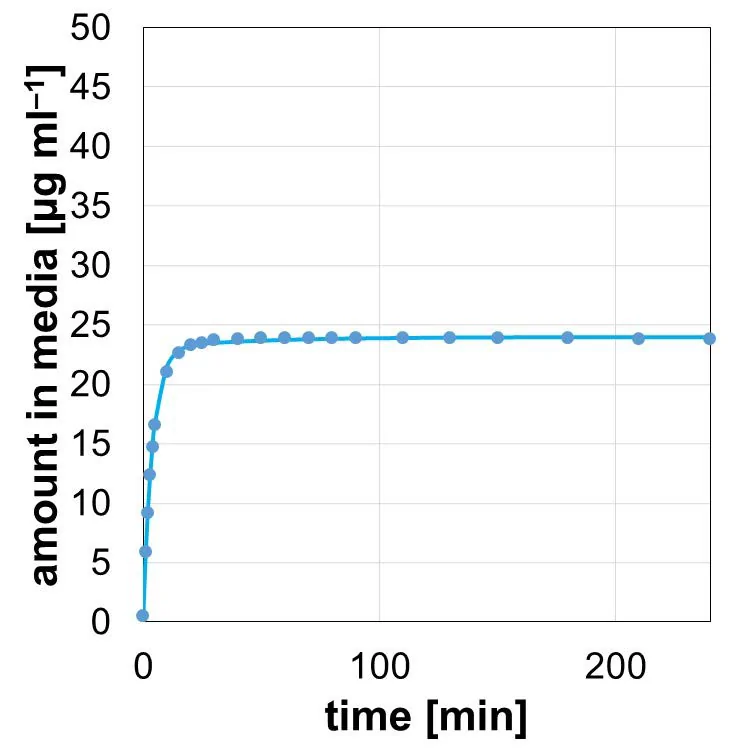
Dissolution of amorphous indomethacin in the presence of 250 μg/mL dissolved PVP at 25 °C. Indomethacin (*t* = 0) = 250 µg ml^−1^, *k*_1_ = 100 min^–1^, *k*_2_ = 0.024 min^–1^, *k*_3_ = 0.023 min^–1^, *k*_4_ = 0.024 min^–1^.

**Figure 6:**
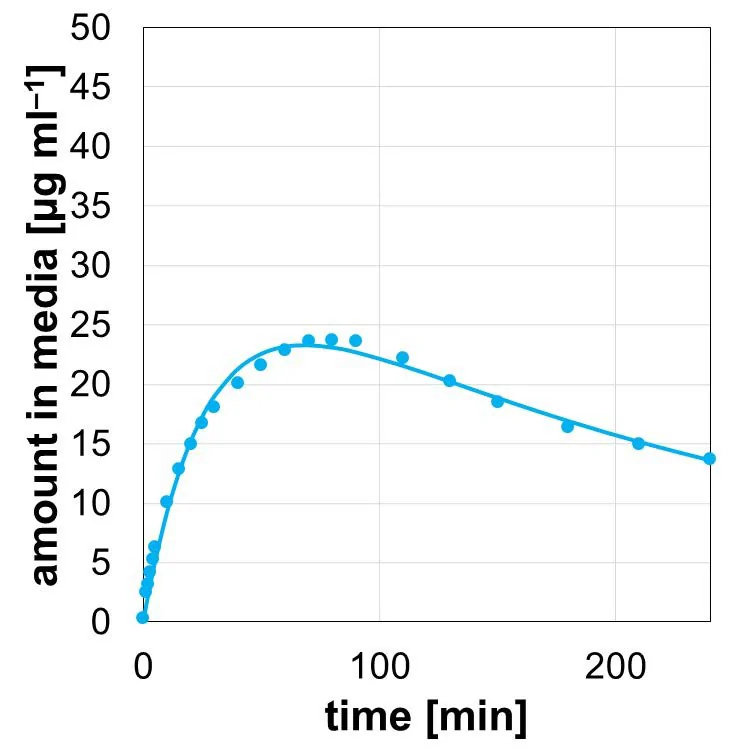
Dissolution of amorphous indomethacin in the presence of 250 μg/mL dissolved PVP at 37 °C. Indomethacin (*t* = 0) = 250 µg ml^–1^, k_1_ = 100 min^–1^, k_2_ = 0.0044 min^–1^, k_3_ = 0.036 min^–1^, k_4_ = 0.00039 min^–1^.

**Figure 7:**
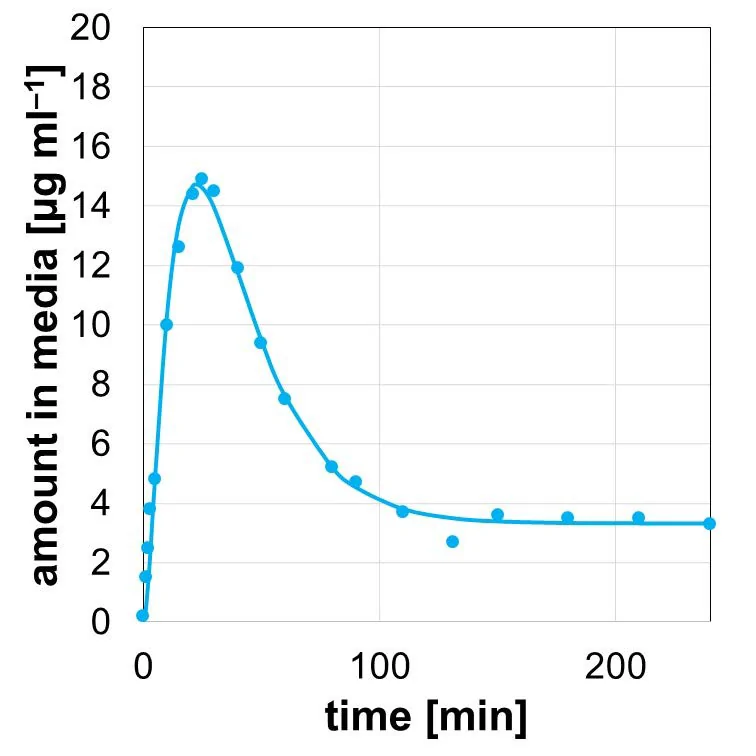
Dissolution of amorphous indomethacin at 25 °C. Indomethacin (*t* = 0) = 250 µg ml^−1^, k_1_ = 0.056 min^–1^, k_2_ = 0.056 min^–1^, k_3_ = 0.37 min^–1^, k_4_ = 0.0049 min^–1^.

**Figure 8:**
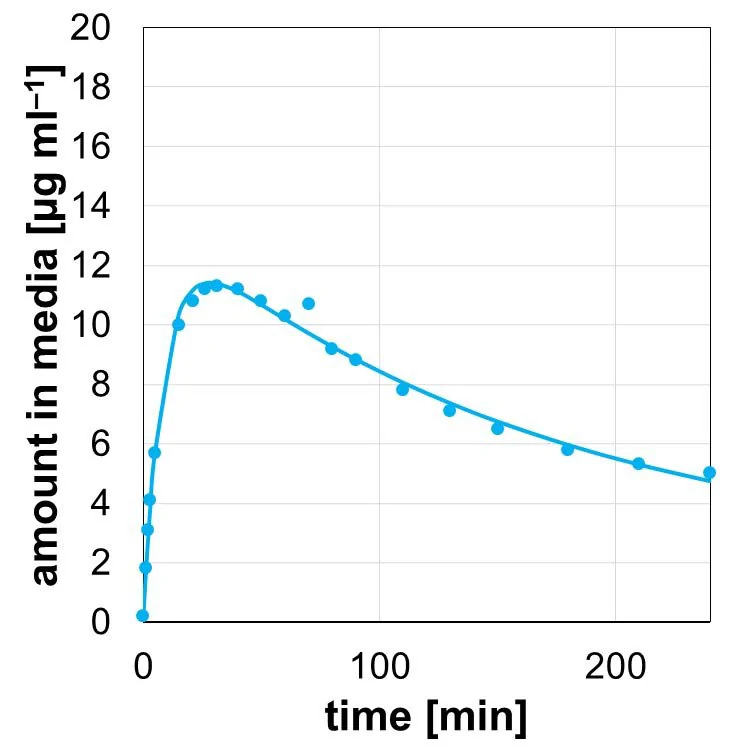
Dissolution of amorphous indomethacin at 37 °C. Indomethacin (*t* = 0) = 250 µg ml^−1^, k_1_ = 100 min^–1^, k_2_ = 0.006 min^–1^, k_3_ = 0.114 min^–1^, k_4_ = 0.00091 min^–1^.

**Figure 9:**
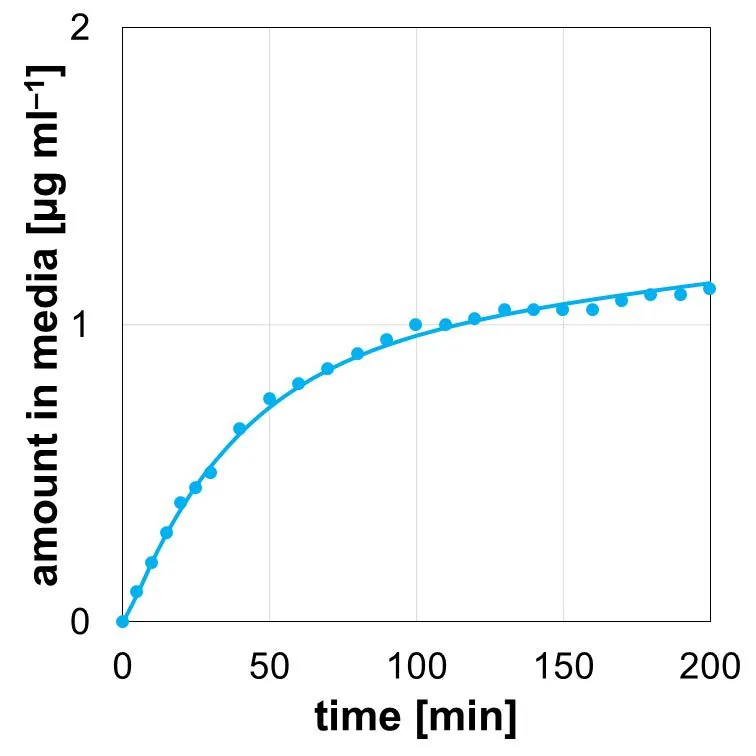
Dissolution of crystalline at 37 °C as a reference. felodipine (*t* = 0) = 500 µg ml^−1^, k_1_ = 1.0 min^–1^, k_2_ = 0.000051 min^–1^, k_3_ = 0.025 min^–1^, k_4_ = 0.0013 min^–1^.

**Figure 10:**
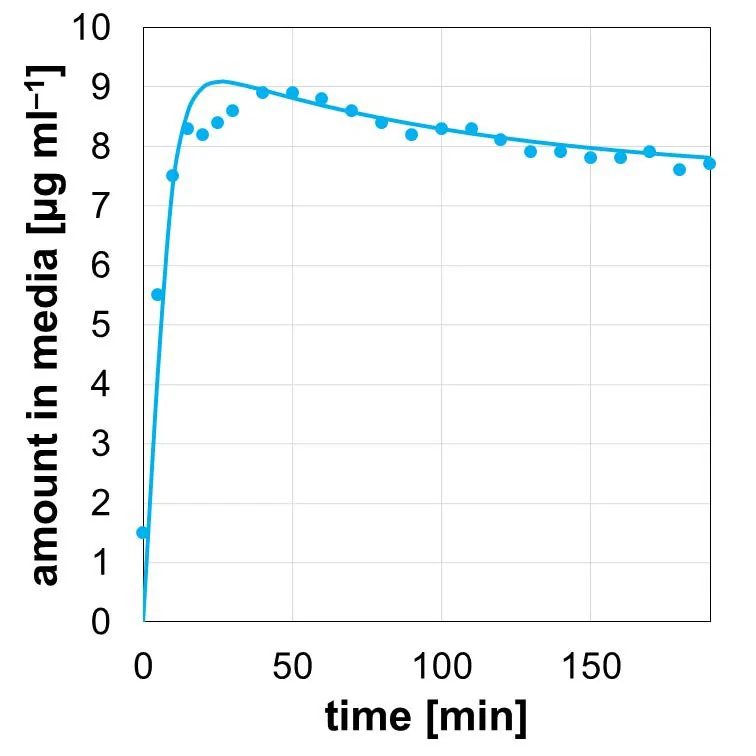
Dissolution of amorphous felodipine at 37 °C in the presence of various polymers pre-dissolved. felodipine (*t* = 0) = 500 µg ml^−1^, k_1_ = 0.2 min^–1^, k_2_ = 0.0098 min^–1^, k_3_ = 0.506 min^–1^, k_4_ = 0.0077 min^–1^.

**Figure 11:**
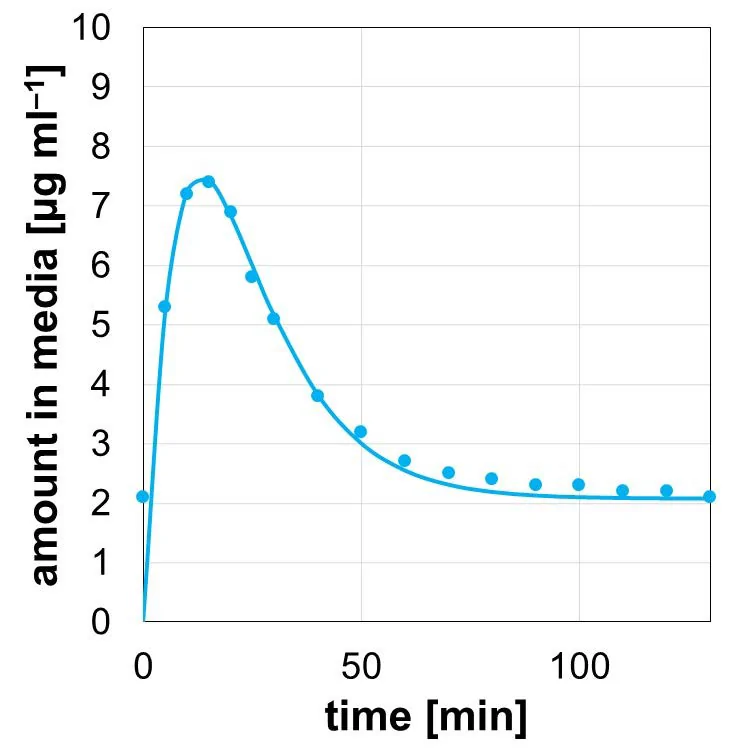
Dissolution of amorphous felodipine at 37 °C in the presence of various polymers pre-dissolved. felodipine (*t* = 0) = 500 µg ml^−1^, k_1_ = 0.1 min^–1^, k_2_ = 0.077 min^–1^, k_3_ = 2.31 min^–1^, k_4_ = 0.0096 min^–1^.

**Figure 12:**
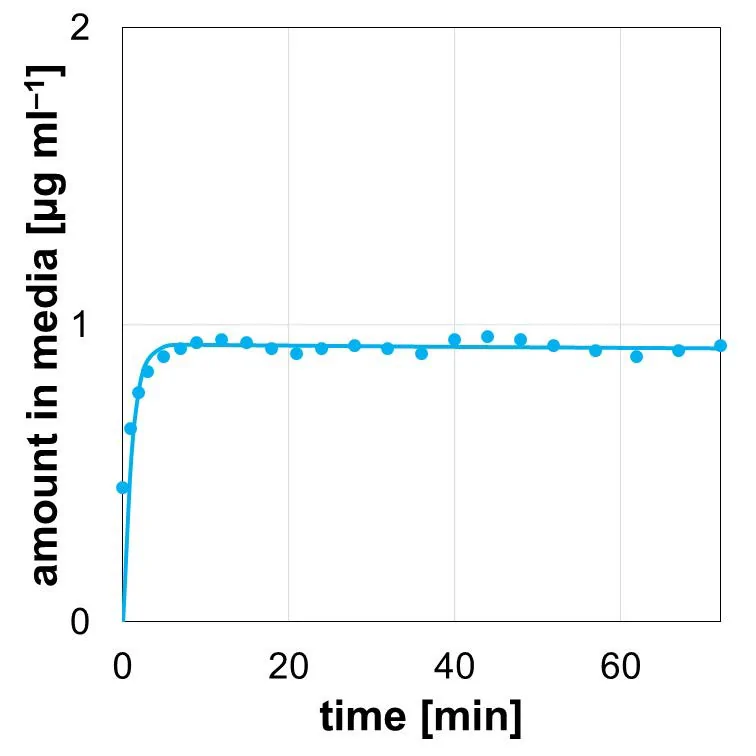
Dissolution of amorphous felodipine, where the amount of solid added per mL of dissolution medium was varied. felodipine (*t* = 0) = 1000 µg ml^−1^, k_1_ = 1.0 min^–1^, k_2_ = 0.01 min^–1^, k_3_ = 10.98 min^–1^, k_4_ = 0.00998 min^–1^.

**Figure 13:**
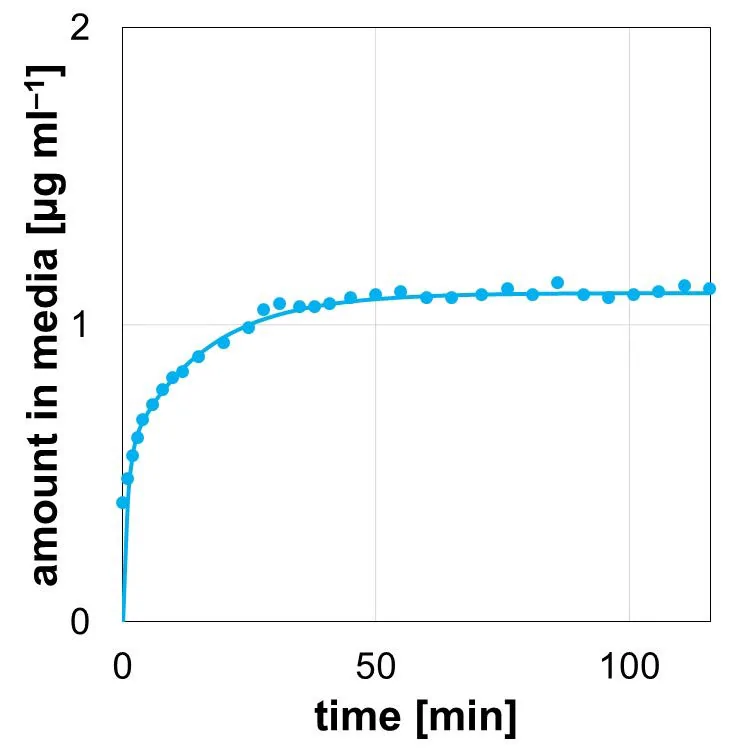
Dissolution of amorphous felodipine, where the amount of solid added per mL of dissolution medium was varied at 25 °C. felodipine (*t* = 0) = 500 µg ml^−1^, k_1_ = 1.2 min^–1^, k_2_ = 0.066 min^–1^, k_3_ = 56.28 min^–1^, k_4_ = 0.12 min^–1^.

**Figure 14:**
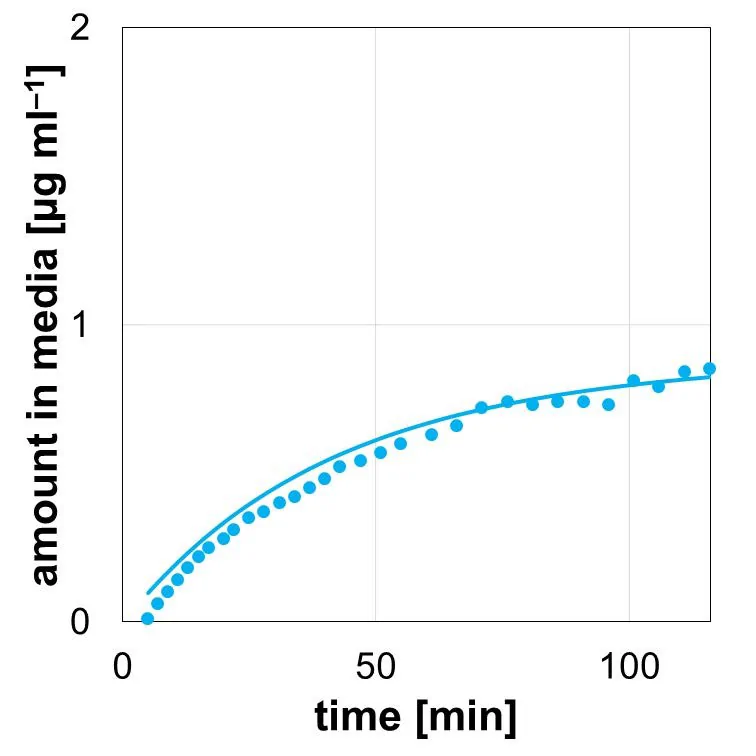
Dissolution of amorphous felodipine, where the amount of solid added per mL of dissolution medium was varied at 37 °C. felodipine (*t* = 0) = 100 µg ml^−1^, k_1_ = 5.0 min^–1^, k_2_ = 0.000214 min^–1^, k_3_ = 0.025 min^–1^, k_4_ = 0.00062 min^–1^.

**Figure 15:**
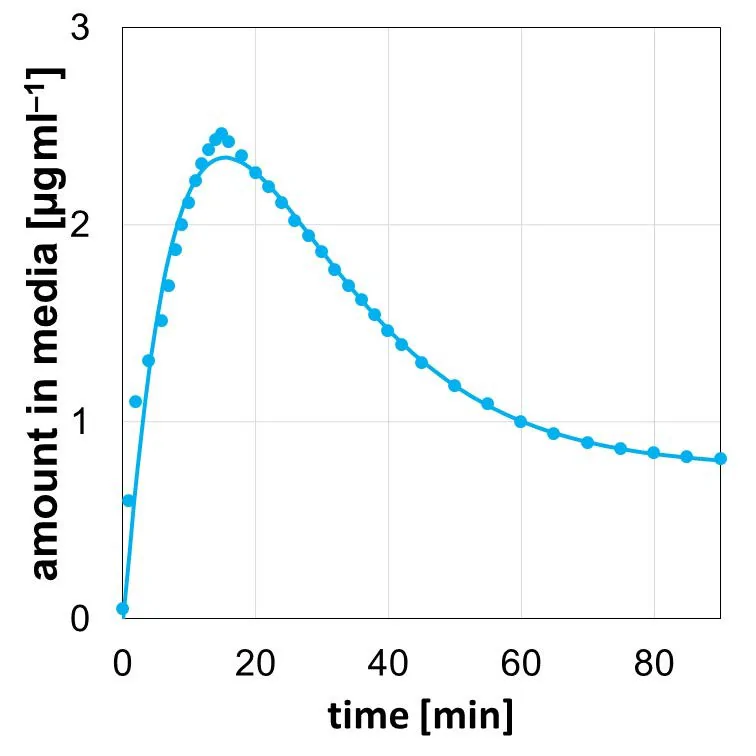
Dissolution of amorphous felodipine, where the amount of solid added per mL of dissolution medium was varied at 25 °C. felodipine (*t* = 0) = 500 µg ml^−1^, k_1_ = 0.08 min^–1^, k_2_ = 0.071 min^–1^, k_3_ = 6.56 min^–1^, k_4_ = 0.01 min^–1^.

As illustrated in Figure 5, Figure 6, Figure 7, Figure 8, Figure 9, Figure 10, Figure 11, Figure 12, Figure 13, Figure 14 and Figure 15, the presented kinetic model consistently demonstrates excellent agreement with macroscopic concentration–time traces. The framework successfully captures monotonic saturation plateaus, delayed release patterns, and transient supersaturation profiles characterized by pronounced “spring and parachute” behavior. Notably, variations in polymer-mediated crystallization inhibition, thermal effects, and dose strength are effectively separated into distinct shifts among the rate constants (*k*_1_–*k*_4_).

Rapid initial solubilization is reflected in high *k*_1_ values, whereas mass transfer into the bulk dissolution medium (*k*_2_) dictates the steepness of the initial concentration rise.

The apparent global crystallization constant (*k*_3_) quantifies the rate of precipitation into the stable crystalline phase. In systems where polymer inhibitors (e.g., HPMC or PVP) effectively sustain supersaturation, *k*_3_ remains relatively low compared to *k*_2_, preserving a broad concentration peak. Conversely, under high initial drug loadings where rapid nucleation dominates, *k*_3_ is significantly higher, driving de-supersaturation toward the equilibrium solubility limit.

These results confirm that the proposed model provides a reliable, operational tool for quantifying drug release dynamics across highly diverse formulations without requiring explicit micro-structural or molecular-level parameterization.

To ensure meaningful and physically sound comparisons across product batches or formulation variants, the model should be parameterized by keeping as many shared system constants fixed as possible and optimizing only the minimum number of parameters necessary to describe the observed dissolution behavior. Although parameter uncertainty can be assessed using statistical methods, including F-tests, t-tests, and Mahalanobis distance analysis, minimizing the number of freely fitted parameters remains essential for ensuring the numerical stability and reliability of the estimated rate constants. Avoiding over-parameterization reduces the risk of overfitting and helps preserve the model’s ability to detect genuine formulation-dependent differences, thereby providing a robust foundation for subsequent comparative statistical analyses, as detailed in Section 7.

Comparison of different pharmaceutical formulation variants is possible, but requires caution when, e.g., the applied polymer is changed. Differences in *k*_1_, *k*_2_, and the initial crystalline fraction ([AC]_0_) can quantify differences in hydration/wetting, drug diffusion, and initial solid-state properties. However, changing the polymer may also affect precipitation kinetics; thus, *k*_3_ and *k*_4_ cannot necessarily be assumed constant. The model should therefore be regarded as a mechanistic framework for comparing batches or formulations where systematic alterations are expected.

Comparison across production batches is necessary for these pharmaceutical formulations, because amorphous solid dispersions are metastable systems, and processing or storage variations may alter their drug dissolution and release properties. Changes in the fitted kinetic parameters may therefore provide a mechanistically informed measure of batch-to-batch consistency beyond the standard analysis of the dissolution profiles alone.

## 7. Modeling the Dissolution Profiles of Beclomethasone Dipropionate from Alginate Aerogels

A study by Duong et al. focuses on the preparation, characterization, and drug release behavior of alginate aerogels impregnated with beclomethasone dipropionate (BDP), a poorly soluble corticosteroid administered pulmonary [4,13]. The aerogels were synthesized using a sol–gel method, followed by supercritical drying [17], and subsequently loaded with BDP through a supercritical impregnation technique [9,28,36]. Dissolution studies were conducted using two different initial drug loadings [37].

**Figure 16:**
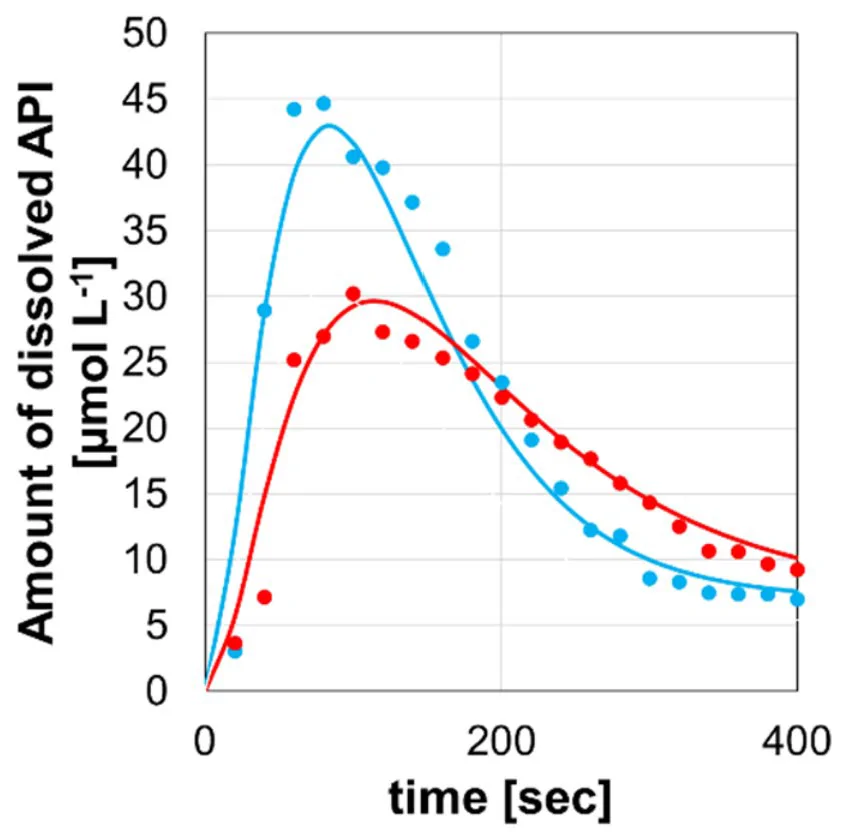
Drug release from an experimental aerogel formulation. The blue dots represent the experimental concentrations obtained for the higher strength formulation, while the blue line shows the fitted model curve. The red dots and red line correspond to the lower strength formulation and its fitted model, respectively. Both formulations were modeled using identical kinetic parameters (*k*_1_ = 0.018 s^–1^, *k*_2_ = 0.040 s^–1^, *k*_3_ = 0.021 s^–1^, *k*_4_ = 0.0011 s^–1^, S = 7.0 µM), with only the initial API amount ([AT]_0_) differing. [AT]_0_A = 140 µM, [AT]_0_B = 60 µM.

These dissolution curves were fitted using the approach presented in this paper under the specific conditions. The only difference is the initial amount of the API in the aerogel formulations. The simulated dissolution curves closely resembled the experimental results (Figure 16).

The model accurately describes the initial acceleration phase observed at the start of the dissolution process. Introducing a lag time in this model is evidently unnecessary.

The model also successfully demonstrates the intersection point of the dissolution curves. This confirms that, despite starting with a lower initial amount of API, higher concentrations can be achieved at certain time points. This behavior could be promising for certain therapeutic applications, as it may offer an improved therapeutic-effect-to-side-effect ratio, thereby aiding in the design of optimized pharmaceutical formulations.

## 8. Determining Biowaiver in Relation to the Spring Effect

A biowaiver refers to the regulatory allowance that exempts a pharmaceutical company from conducting in vivo bioequivalence studies for a generic drug product [28,38,39]. This waiver is typically granted when certain criteria are met, such as rapid dissolution, linear pharmacokinetics, or classification within specific Biopharmaceutics Classification System (BCS) categories [1,2,3,28,39]. The rationale behind a biowaiver is that, under these circumstances, in vivo studies may not provide significant additional value, as the pharmacokinetic behavior of the drug is unlikely to differ meaningfully from that of the reference product [28,39].

In the context of biowaiver evaluation, it is essential to understand the distinction between strength and label claim as defined in the Section “Relationship Between Initial Conditions and Physical Processes” [28,39].

When assessing biowaiver eligibility—especially for formulations exhibiting complex release behaviors such as the spring effect—careful consideration must be given not only to the dissolution profile itself, but also to the impact of varying strengths and their corresponding label claims on release kinetics. These distinctions play a critical role in determining whether two formulations can be deemed bioequivalent in the absence of in vivo data [12,13,14,15,16].

While routine quality control (QC) dissolution tests for immediate-release forms strictly demand sink conditions to ensure 100% release, evaluating advanced supersaturating drug delivery systems—such ASDs—under non-sink or near-non-sink conditions is crucial within a Quality by Design (QbD) paradigm. Sink conditions conceal kinetic phenomena such as rapid supersaturation (“spring”) and subsequent precipitation (“parachute”), which directly dictate the in vivo bioavailable concentration profile of metastable amorphous formulations.

The proposed kinetic model bridges routine macro-level dissolution data and formulation science by establishing a mechanistic relationship between macroscopic concentration–time trajectories and underlying rate constants (*k*_1_ to *k*_4_). In a QbD framework, these fitted rate constants serve as sensitive critical quality attributes (CQAs). While standard single-point QC specifications (e.g., *Q* > 80% at 45 min) cannot detect early precipitation or subtle matrix degradation, tracking variations in *k*_1_ (hydration) and *k*_3_ (precipitation) provides an advanced diagnostic tool for batch-to-batch consistency evaluations. Furthermore, the model explicitly incorporates the nominal label claim as a normalization baseline. This allows manufacturers to separate kinetic differences caused by API solid-state aging (e.g., partial recrystallization during storage) from simple dose-scaling artifacts. Consequently, the presented approach can extend regulatory control by supplying a robust, parameter-based specification engine capable of verifying matrix stability and ensuring batch comparability across the product lifecycle.

For products exhibiting the spring effect, model-independent approaches (such as *f*1, *f*2, and the multivariate confidence region) are inadequate for assessing biowaiver eligibility for several reasons. When comparing products with identical formulations but different label-claim strengths, significant differences are observed in their dissolution profiles. The higher the strength, the faster the dissolution process, along with the formation of the thermodynamically stable crystalline form [5,12,13,17,23]. A striking indication of this phenomenon is the substantial variation in *c*_max_ and the fact that the dissolution curves of different strengths intersect in the declining concentration phase of the dissolution [12,13,14,15,16].

**Figure 17:**
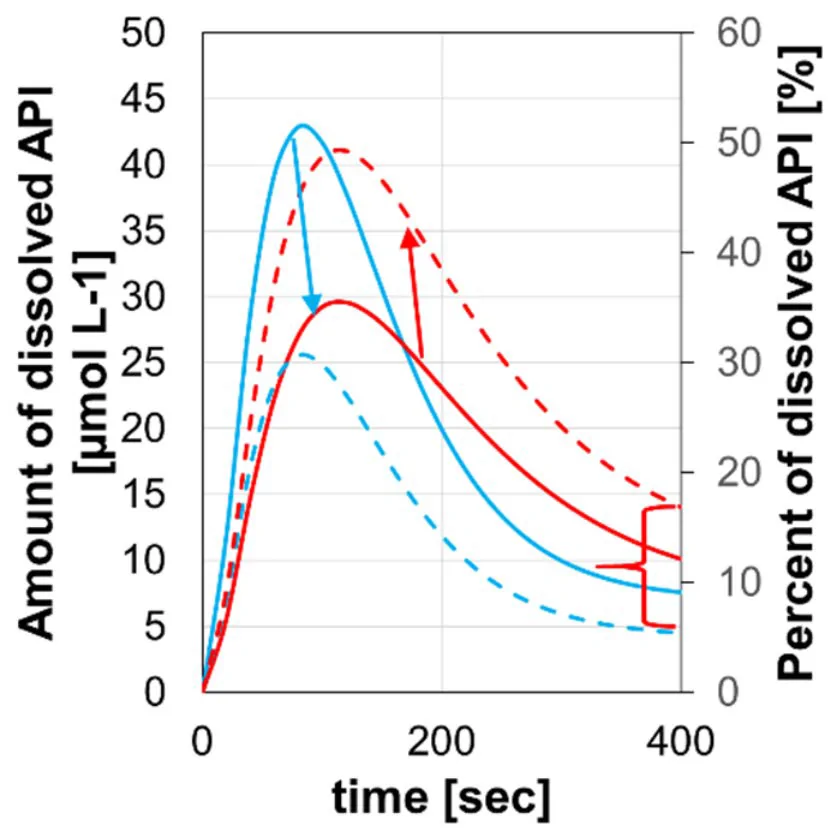
Changes in drug release profiles due to conversion to percentage of dissolved API. Blue solid line: dissolved amount in the case of higher strength; blue dashed line: dissolved amount normalized to the label claim in the case of higher strength. Red solid line: dissolved amount in the case of lower strength; red dashed line: dissolved amount normalized to the label claim in the case of lower strength.

The difference in *c*_max_ cannot be eliminated by normalizing the dissolution results to the label claim percentage, and in such cases, the final dissolution value will not be identical, despite both dissolution profiles converging to the same concentration at the end of the process (Figure 17) [8,13,18].

On a percentage scale, the two dissolution curves are inverted. While the dissolution curve for the higher-dose experiment runs higher on an absolute mass scale compared to the lower-dose dissolution, it appears significantly lower on the relative percentage scale than the lower-dose experiment. This can be misleading if the kinetic behavior is not taken into account [12,13,14,15,16].

## 9. Proposed Strategy for Establishing Similarity

Extending the kinetic approach to biowaiver assessments and dissolution profile similarity is scientifically justified, as traditional model-independent metrics, such as the *f*_2_ similarity factor, may be inadequate for non-monotonic dissolution profiles characterized by supersaturation. For formulations exhibiting a pronounced “spring effect,” comparing dissolution profiles across different dosage strengths on a percentage basis can introduce mathematical artifacts that obscure the underlying release behavior. Under non-sink conditions, higher-strength formulations may exhibit lower percentage-based dissolved concentrations because of more rapid precipitation, potentially masking genuine formulation equivalence.

The proposed framework addresses this limitation by distinguishing formulation strength, represented by the initial total concentration ([AT]_0_), from the intrinsic kinetic parameters (*k*_1_, *k*_2_, and *k*_3_). When assessing biowaiver eligibility across different dosage strengths or following minor modifications to the matrix composition, [AT]_0_ is treated as a fixed physical input, whereas similarity is assessed using the fitted rate constants. Multivariate statistical methods, such as Mahalanobis distance analysis, or statistical procedures for comparing rate constants, including variance-homogeneity testing (F-test) followed by a two-sample t-test, can then be applied to determine whether the formulations exhibit comparable dissolution and de-supersaturation kinetics.

Although biowaiver assessment and supersaturation characterization have traditionally served distinct purposes, both are governed by the underlying dissolution–precipitation processes of the polymer-matrix system. Accordingly, normalizing dissolution profiles to the label claim and comparing the confidence regions of the fitted kinetic parameters may provide a more mechanistically informative and statistically robust framework for biowaiver assessment. This approach offers a potential alternative to conventional percentage-based *f*_2_ evaluations, which may be unsuitable for complex amorphous formulations exhibiting non-monotonic dissolution behavior.

Given these considerations, only *k*_1_, *k*_2_, and *k*_3_ are subjected to statistical evaluation, which can be performed using multiple approaches. The analysis formulates the following statistical hypotheses: the null hypothesis (H_0_) states that there is no significant difference between the fitted *k*_1_ and *k*_2_ (or *k*_3_) values, whereas the alternative hypothesis (H_1_) assumes a significant difference exists. Acceptance criteria can be established via two alternative routes. The first approach relies on calculating the Mahalanobis distance [40]. If the resulting distance is smaller than the critical threshold (e.g., within the 90% confidence region where *p* = 0.9), the profiles can be considered statistically similar. The second, simpler method involves an F-test to assess variance homogeneity (if *p* > 0.05), followed by a standard two-sample Student’s t-test or Welch’s t-test depending on variance equality. A non-significant t-test result confirms that dissolution profiles match in terms of kinetic parameters.

If the Mahalanobis distance of the fitted *k*_1_ and *k*_3_ parameters from 12-12 test runs falls within the acceptance criteria of the multivariate confidence region, or if the F-test and subsequent t-test confirm no significant differences, the dissolution profiles of different label-claim strengths can be considered similar. This approach could indeed be a viable alternative to traditional model-independent methods for regulatory applications, particularly in cases where the dissolution mechanism is governed by complex phase transitions rather than simple diffusion-driven release [12,13,14,15,16].

### Presenting Significant Difference Between k_2_ of Different Label Claims

**Figure 18:**
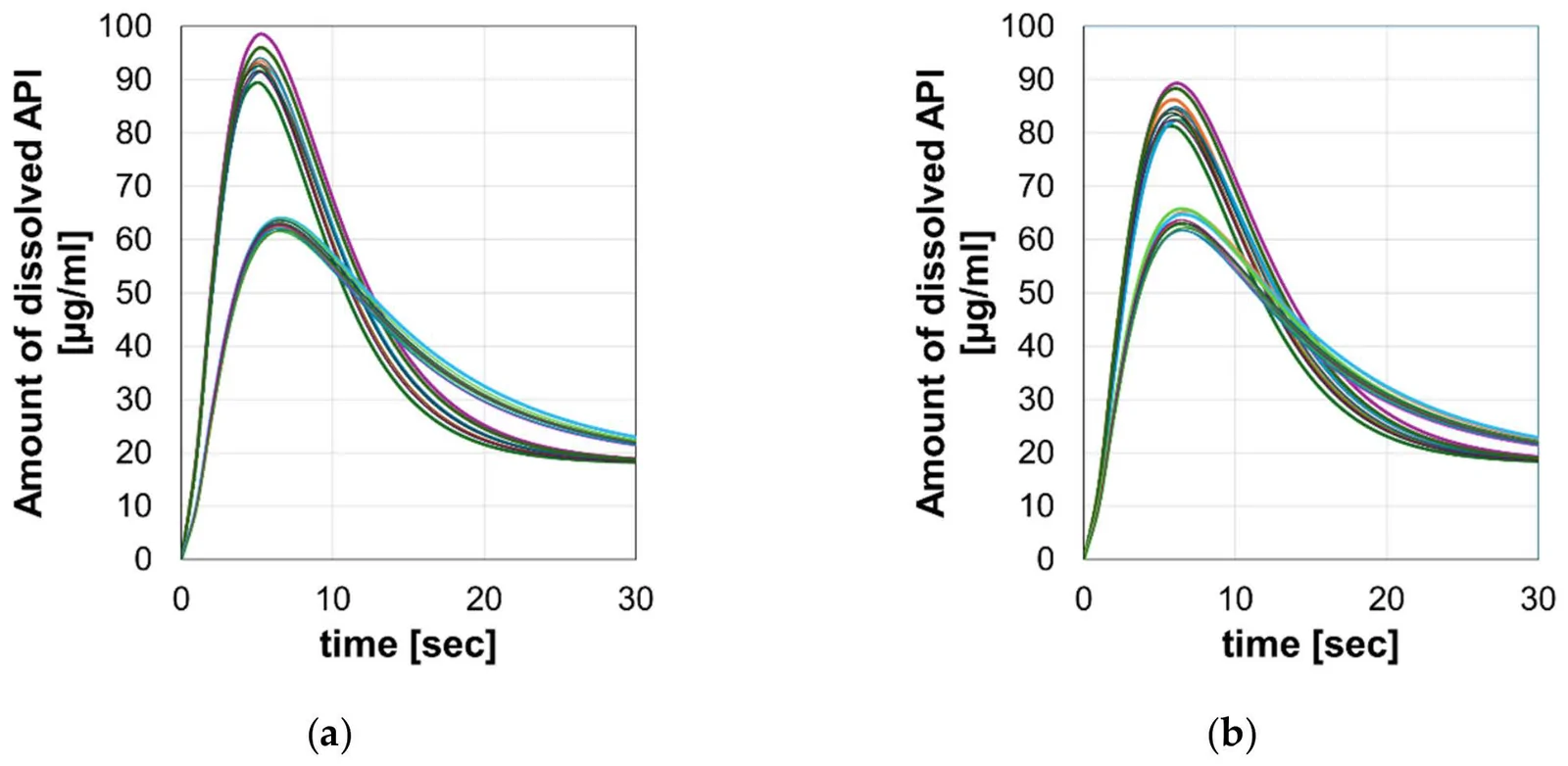
Simulated dissolution profiles illustrating the impact of varying *k*_2_ values, showing negligible differences in panel (**a**) AT(0)(high) = 200 µg/mL, *k*_1_ = 0.50 s^–1^, *k*_2_ = 0.59 s^–1^, AT(0)(low) = 100 µg/mL, *k*_1_ = 0.50 s^–1^, *k*_2_ = 0.60 s^–1^; and pronounced divergence in panel (**b**) AT(0)(high) = 200 µg/mL, *k*_1_ = 0.50 s^–1^, *k*_2_ = 0.39 s^–1^, AT(0)(low) = 100 µg/mL, *k*_1_ = 0.50 s^–1^, *k*_2_ = 0.60 s^–1^.

It can be observed that solely examining the experimental data does not allow for a definitive conclusion on whether the *k*_1_ and *k*_2_ values are similar. Figure 18 illustrates how a 0.2 unit increase in *k_2_* for the higher strength formulation results in a modest shift in the dissolution profile. Specifically, *t*_max_ appears to occur earlier, and the peak concentration increases slightly compared to the lower strength formulation. However, the maximum concentration remains below 100 µg/mL in both cases, and the visual difference is subtle. Therefore, statistical evaluation is necessary to determine whether the observed variation in *k_2_* is significant or not.

Consequently, future specifications should be established based on kinetic parameters rather than solely on the amount of drug dissolved at predefined time points, because the dissolution profiles of different label-claim strengths diverge to such an extent that neither absolute nor percentage-based dissolution results allow for a meaningful comparison. In these cases, model fitting and the subsequent comparison of kinetic parameters become indispensable for establishing true similarity.

## 10. Example of Model-Dependent Approach for Biowaiver by Distance

A model dataset was generated that presents the dissolution results for two different dose strengths (Figure 18). The kinetic profiles differ only in [AT]_0_, incorporating variability, while the *k*_1_ and *k*_2_ parameters were modeled with the same expected value and a small variance. The *k*_4_ parameter was set as a constant considering solubility, whereas k_3_ was fitted.

During the analysis, only *k*_1_, *k*_2_, and *k*_3_ needed to be fitted. To assess biowaiver eligibility, only the Mahalanobis distance of the fitted *k*_1_ and *k*_2_ parameters was evaluated [38].

Statistical hypothesis:

**H0:**  *There is no significant difference between fitted k_1_ values and fitted k_2_ values.*

**H1:**  *There is significant difference between fitted k_1_ values and fitted k_2_ values.*

In the presented case, the Mahalanobis distance (Upper_90_CR_MSD = 13.24) exceeds the critical threshold (Max_MSD = 11.82), indicating a statistically significant difference between the fitted *k*_1_ and *k*_2_ parameters of the two dissolution profiles (Figure 18).

For biowaiver analysis, the determination of the pooled sample Mahalanobis distance of the fitted parameters is essential, as a simple comparison of dissolution curves is insufficient for detecting underlying differences [28,38]. The dissolution profile is quantitatively characterized by the rate constants. Consequently, the identification of behavioral differences, if present, is not feasible without a model-based approach [12,13,14,15,16].

An alternative methodology requiring fewer mathematical computations also demonstrates differences between the fitted parameters. Performing an F-test on the fitted *k*_2_ values, followed by a two-sample Student’s t-test, results in a low *p*-value (*p* << 0.01). Therefore, the fitted *k*_2_ values exhibit a statistically significant difference across different dosage strengths.

The presented evaluation highlights the necessity of advanced statistical methods in dissolution testing, reinforcing the importance of kinetic parameter-based assessments in pharmaceutical formulation development and regulatory decision-making [28,38,40,41].

## 11. Conclusions

This study presents a model-dependent approach for evaluating the dissolution behavior of formulations exhibiting the spring effect. Traditional model-independent methods often fail to capture the complexity of such dissolution processes, particularly when significant differences in kinetic parameters exist despite similar overall dissolution profiles. By developing a kinetic model that accounts for hydration, dissolution, and crystallization processes, we demonstrated that dissolution profiles of different label-claim strengths can be quantitatively compared beyond simple percentage-based analysis.

The results indicate that solely analyzing dissolution data without kinetic modeling does not provide sufficient insight into whether key dissolution parameters remain comparable across different strengths. The proposed model enables a more precise assessment by fitting dissolution curves under controlled assumptions and using Mahalanobis distance to establish statistical similarity. Furthermore, complementary statistical tests, including the F-test and two-sample t-test, further validate the comparability of the fitted kinetic parameters.

Beyond its application in bioequivalence and biowaiver assessments, this modeling approach has broader implications for pharmaceutical development, manufacturing, and quality control. The findings suggest that future regulatory specifications should shift from dissolution percentage-based limits toward kinetic parameter-based acceptance criteria. Implementing model-based evaluations in routine stability studies could provide a more robust method for detecting formulation changes over time, ensuring batch-to-batch consistency and product performance throughout the product lifecycle.

Although the proposed model does not resolve molecular-level mechanisms, it provides a practical and scalable approach for formulation assessment, bridging the gap between empirical dissolution data and regulatory decision-making.

## Data Availability

The data that support the findings of this study are available from the corresponding author upon reasonable request.
